# Extensive abdominal wall ulceration as a late manifestation of antiphospholipid syndrome: a case report

**DOI:** 10.1186/s13256-018-1753-5

**Published:** 2018-08-14

**Authors:** Yogesh Sharma, Karen Humphreys, Campbell Thompson

**Affiliations:** 1 Department of General Medicine, Flinders Medical Centre, Flinders Drive, Bedford Park, Adelaide, South Australia Australia; 2 College of Medicine and Public Health, Flinders University, Flinders Drive, Bedford Park, Adelaide, South Australia Australia; 3 Karen Humphreys, Flinders University, Flinders Drive, Bedford Park, Adelaide, South Australia Australia; 4 Discipline of Medicine, University of Adelaide, Adelaide, South Australia Australia

**Keywords:** Antiphospholipid syndrome, Antiphospholipid antibodies, Systemic lupus erythematosus, Cutaneous ulcers, Anticoagulation

## Abstract

**Background:**

Antiphospholipid syndrome is an autoimmune disorder characterized by the presence of antiphospholipid antibodies and commonly presents with vascular thromboembolic phenomena, thrombocytopenia, and obstetric complications. Antiphospholipid syndrome can be classified as either primary or secondary to other connective tissue diseases. Dermatologic manifestations are common; however, non-vasculitic skin ulceration is an uncommon manifestation of antiphospholipid syndrome with limited treatment options.

**Case presentation:**

In this paper we report the case of a 58-year-old white woman who developed necrotic abdominal wall ulcers 27 years after a diagnosis of secondary antiphospholipid syndrome associated with systemic lupus erythematosus. The ulcers developed despite our patient being on therapeutic anticoagulation with warfarin and were resistant to further increases in the intensity of anticoagulation. Management was further complicated due to reluctance on the part of our patient to switch over to injectable heparin.

**Conclusions:**

This case highlights a rare late dermatologic presentation of antiphospholipid syndrome, which responded poorly to conventional anticoagulation with warfarin. Current management is limited to experimental therapies and the role of newer anticoagulants is still unknown.

## Background

Antiphospholipid syndrome (APS) is an autoimmune condition that is characterized by the presence of antiphospholipid antibodies (aPLs) and is associated with thrombosis, thrombocytopenia, and morbidity during pregnancy [1]. Clinical manifestations of APS can be broad and may include non-criteria manifestations, for example, skin lesions, thrombocytopenia, hemolytic anemia, nephropathy, cardiac valve disease, cognitive dysfunction, and chorea [2]. Other manifestations include: pregnancy morbidity (recurrent fetal loss, preeclampsia, and growth restriction); venous, arterial, or small vessel thrombosis; and catastrophic APS which may lead to multiorgan failure associated with microangiopathy [3].

The major aPLs found in APS include anticardiolipin antibodies (aCLs), lupus anticoagulant (LA), and anti-β2-glycoprotein I antibodies (aβ2GPI) [4]. APLs can be found in 1 to 5% of healthy adults, but in systemic lupus erythematosus (SLE) the prevalence of antibodies is greatly increased and between 50 and 75% of these patients develop thrombotic events consistent with APS [5]. APS can be either primary or secondary. Primary APS occurs in the absence of other autoimmune disorders whereas secondary APS is associated with SLE, rheumatoid arthritis, and other autoimmune conditions. The international Sapporo criteria (1999), revised in 2006, recommend that the diagnosis of APS be considered when at least one of the major aPLs is detected on two or more occasions with a 12-week interval between measurements and is associated with a clinical condition such as thrombosis or morbidity during pregnancy [6].

Dermatologic manifestations are common (49% of patients) and livedo reticularis is the most common skin finding in patients with APS [7]. A study involving 200 consecutive patients with APS found that the overall prevalence of dermatologic manifestations was similar in primary APS (45%) and SLE-related APS (53%) [7, 8]. Other skin manifestations include cutaneous necrosis, splinter hemorrhages, pyoderma gangrenosum, Raynaud phenomenon, and erythema nodosum [7]. Non-vasculitic skin ulcers are an uncommon manifestation of APS and have been described in 3.5% of cases and are usually located on the extremities while diffuse cutaneous necrosis due to thrombosis of microvasculature can be life threatening and is a therapeutic dilemma with high mortality [9, 10]. Cutaneous necrosis may be the sole presenting feature and may be the only manifestation in 2.5% cases of APS [7].

After a first thrombotic episode in APS, current therapeutic guidelines suggest indefinite anticoagulation with warfarin for secondary prevention to achieve an international normalized ratio (INR) target of between 2.0 and 3.0 [11]. In patients with APS who are anticoagulated with warfarin and develop further thrombosis, the options are to increase the intensity of anticoagulation, to switch over to heparin, or to add aspirin. We describe a clinical report of a rare late skin manifestation of APS due to microthrombosis which developed despite adequate anticoagulation with warfarin and describe the therapeutic limitations in the care of such patients.

## Case presentation

We describe a 58-year-old white woman who was living alone at home with a known history of SLE-associated secondary APS. The diagnosis of SLE had been made 27 years previously when she developed recurrent episodes of pneumonitis, malar rash, and renal failure complicated by recurrent deep vein thrombosis. Blood tests at that time revealed positive antinuclear antibody (ANA), elevated anti-double-stranded deoxyribonucleic acid (anti-dsDNA) titers, and low complement (C3 and C4) levels and urine analysis revealed proteinuria, hematuria, and cellular casts suggestive of lupus nephritis. A diagnosis of secondary APS was made on the basis of positive LA in two determinations with a 3-month interval and a history of recurrent deep vein thrombosis. Additional thrombophilia screening (factor V Leiden mutation, prothrombin gene mutation, factor 8 levels, protein C, protein S, and antithrombin 3) was negative.

At the initial diagnosis, she received pulse cyclophosphamide and prednisolone for lupus nephritis and, after resolution of the initial thrombotic event, she was started on hydroxychloroquine and lifelong warfarin anticoagulation with a target INR between 2 and 3.

Her other medical issues included obesity, obstructive sleep apnea, diet-controlled type 2 diabetes mellitus, hypertension, stage 3B moderate chronic kidney disease, fatty liver, endometrial cancer (treated 14 years ago with progesterone, ongoing surveillance showed no recurrence), left total shoulder joint replacement for severe osteoarthritis, and Sydenham’s chorea.

Her regular medications included warfarin, atenolol, hydroxychloroquine, oxycodone, naloxone, paracetamol, and multivitamins.

Two months prior to the current admission, she developed ulcerative lesions on her anterior abdominal wall which she related to an abdominal ultrasound performed for suspected kidney stones. She reported that, at sites of ultrasound probe pressure, she initially noticed small bruises with subsequent skin breakdown associated with oozing of clear fluid and pain. She consulted her general practitioner who prescribed wound dressings and referred her to our hospital after local wound swabs grew multidrug resistant *Enterobacter cloacae*. At the time of hospital admission she was found to be afebrile and hemodynamically stable and had five ulcers on her anterior abdominal wall. The largest ulcer was located over her right flank and had irregular margins with surrounding erythema with a clean base. She was seen by a surgeon who did not think that the ulcers needed debridement.

### Investigations

**Fig. 1:**
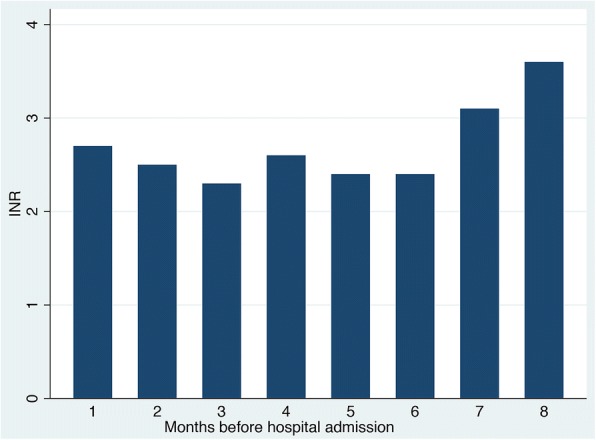
Graph showing international normalized ratio in 8 months prior to hospital admission. *INR* international normalized ratio

Her investigations revealed mild normocytic anemia with stable chronic renal failure, that is, creatinine 134 micromole/L and glomerular filtration rate (GFR) 38 ml/minute; her INR was therapeutic at 2.5. Her INR in the preceding 8 months had been in the therapeutic range of between 2.3 and 3.6 (Fig. 1). Her inflammatory markers were elevated with a C-reactive protein (CRP) of 250 mg/L. A wound swab grew *Enterobacter cloacae*, sensitive to ciprofloxacin, and this was prescribed orally along with local wound dressings. She was discharged home with an instruction to continue warfarin and antibiotics for a period of 2 weeks.

**Fig. 2:**
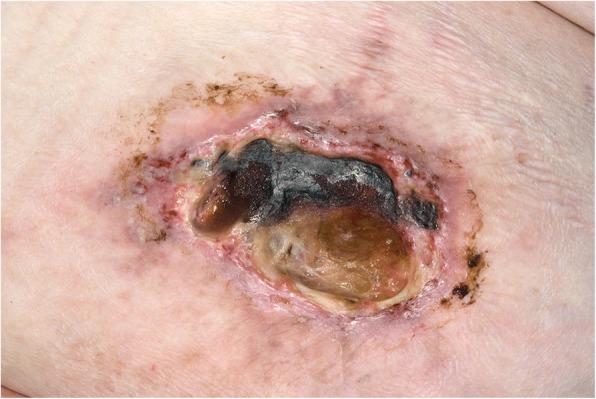
A large necrotic ulcer measuring 11 × 7 cm on anterior abdominal wall showing irregular margins, black eschar, and yellow exudate

**Fig. 3:**
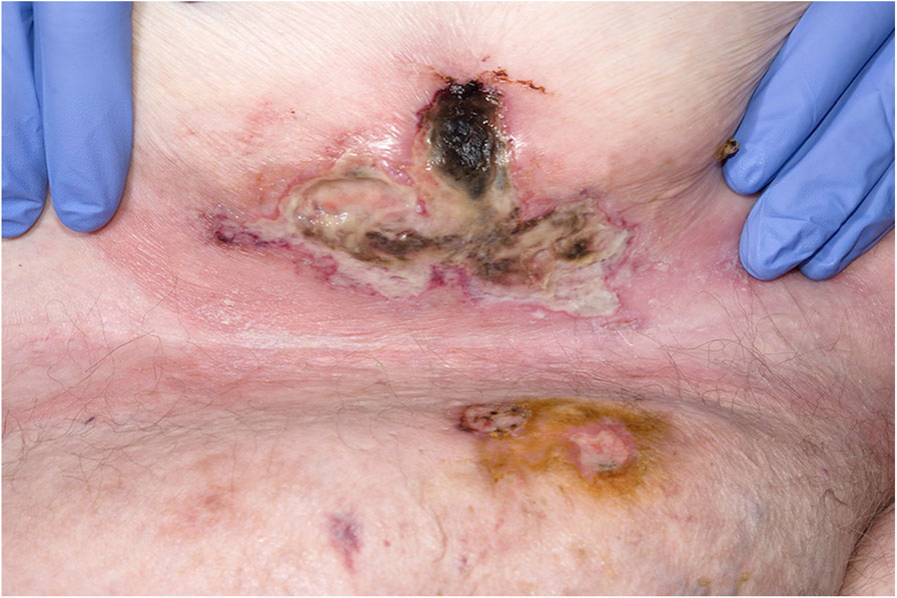
Two smaller ulcers on the paraumbilical and suprapubic regions of abdomen

She re-presented 11 days later with worsening of her symptoms: specifically non-healing ulcers and increasing pain. An examination revealed progression of the abdominal ulcers (Figs. 2 and 3) with the largest ulcer located over her right flank showing increased size and depth, hemorrhagic scab, black eschar, and yellow exudate (Fig. 2). There were new ulcers located on her left breast and left interscapular area. Wound swabs from the abdominal ulcers grew methicillin-resistant *Staphylococci* but repeated blood cultures remained sterile. Her repeat investigations revealed elevation of her white cell count and a further increase in CRP with a mild worsening of anemia but stable renal function. She was found to be positive for LA, but negative for cardiolipin IgG, anti-β2-glycoprotein 1, cryoglobulin, cryofibrinogen, ANA as determined by indirect immunofluorescence (IIF) assay, extractable nuclear antigen (ENA) determined using enzyme-linked immunosorbent assay (ELISA), and anti-neutrophil cytoplasmic antibody (ANCA) but her complement levels were mildly elevated: complement C3 1.93 g/L (normal range 0.85–1.60 g/L) and complement C4 0.44 g/L (normal range 0.12–0.36 g/L). She had a negative hepatitis C screen and urine analysis revealed mild albuminuria with no active sediment on microscopic examination: albumin/creatinine ratio 3.7 (normal < 3.5 mg/mmol).

### Histopathological findings

**Fig. 4:**
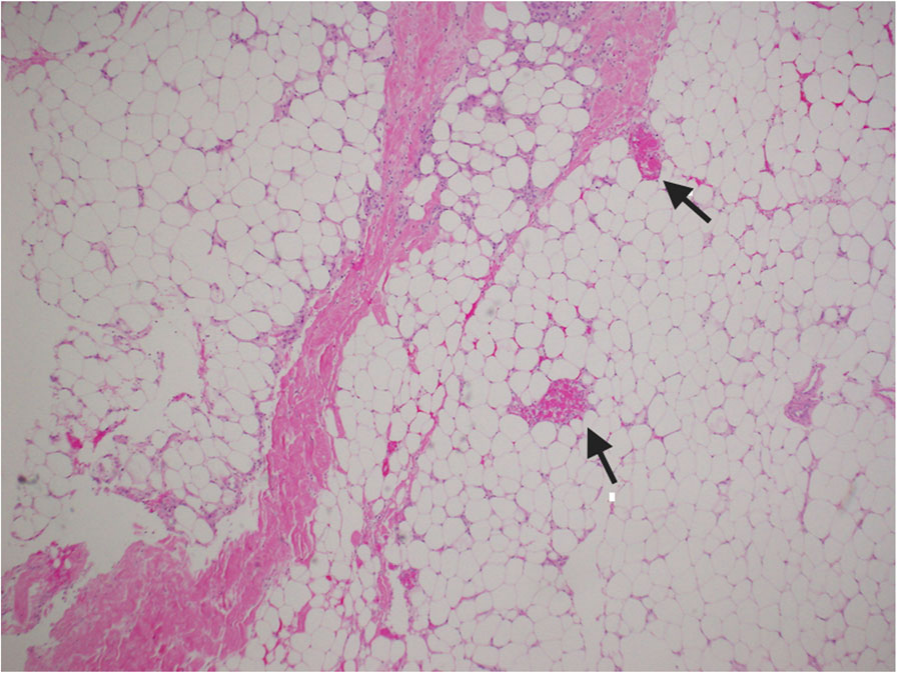
Skin biopsy, light microscopy, hematoxylin and eosin. Histopathological examination revealing fibrin thrombi with no evidence of vasculitis or calciphylaxis. The arrows are pointing to fibrin thrombi within blood vessels

**Fig. 5:**
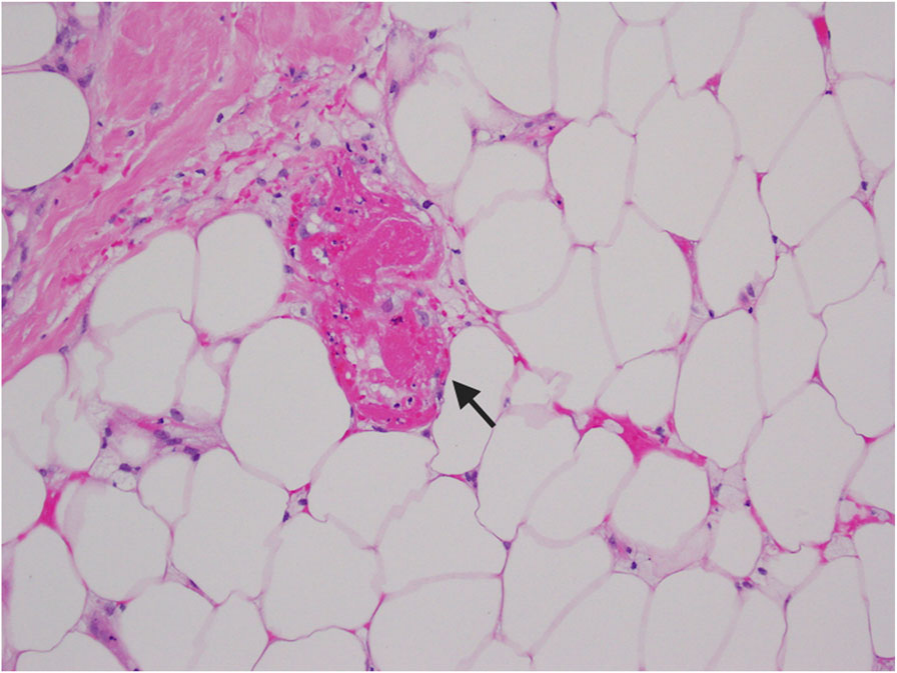
Skin biopsy, hematoxylin and eosin, light microscopy. Fibrin thrombus within a blood vessel. The arrow is pointing to a fibrin thrombus within a blood vessel

A skin biopsy of one abdominal ulcer showed ischemia and necrosis of the subcutaneous fat, with granulation tissue reaction within the dermis and cutaneous necrosis. There was evidence of thrombo-occlusive material (Fig. 4) but there was no evidence of calciphylaxis or medium-sized vessel vasculitis (Fig. 5).

**Fig. 6:**
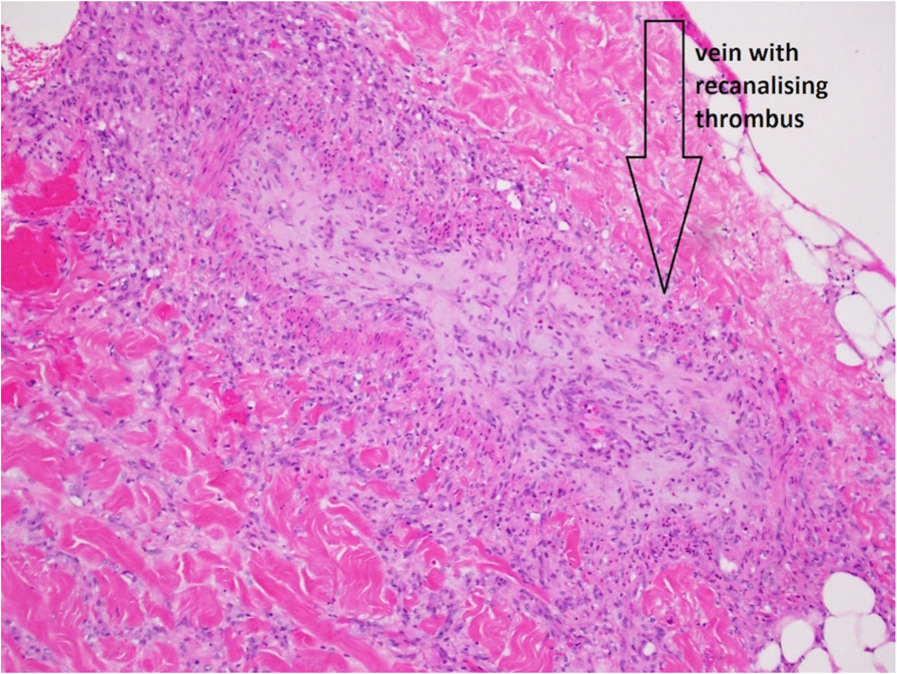
Skin biopsy, hematoxylin and eosin, light microscopy. Vein showing a recanalized thrombus

A second skin biopsy of a right flank ulcer showed numerous microthrombi in small vessels of the subcutaneous fat, including a recanalizing thrombus within a medium-sized vein but no vasculitis (Fig. 6). A note was made of focal fat necrosis with mild inflammation of fat with rare Gram-positive cocci.

### Management

She was commenced on vancomycin and her warfarin anticoagulation was intensified to maintain the INR between 3 and 4 and wound dressings were continued as per consultation with the dermatologists and surgeons. She reported minimal improvement in her symptoms with ongoing pain and non-healing ulcers despite being on high-intensity warfarin anticoagulation and antibiotics.

After a multi-specialty case conference involving the rheumatology, hematology, and dermatology teams, it was thought that her thrombosis was warfarin-resistant and anticoagulation with low molecular weight heparin was suggested but she refused any form of long-term injections. Her clinical course was further complicated by an episode of per rectal bleeding while on high-intensity warfarin anticoagulation and this was managed by blood transfusions. A malignancy screen, including a computed tomography (CT) scan of her chest, abdomen, and pelvis, and a mammogram and pelvic ultrasound excluded underlying occult malignancy as a cause for her hypercoagulable state; a gynecologist excluded recurrence of endometrial cancer through transvaginal ultrasound, hysteroscopy with dilatation and curettage (D & C), and endometrial biopsy.

She was informed that the most likely cause for her symptoms was APS-related skin necrosis with superadded bacterial infection. She remained resistant to the idea of long-term injectable heparin although she was happy to try one of the newer oral direct inhibitors (ODIs) of coagulation despite a lack of firm evidence of efficacy. She was commenced on apixaban with continuation of wound care and an increase in analgesia.

### Follow-up

Changing her anticoagulation from warfarin to apixaban, however, made little difference to her symptoms and she had multiple subsequent admissions with sepsis and bleeding from her ulcers with worsening of renal function. In the subsequent admissions, she was managed with blood transfusions and antibiotics. Plasmapheresis and intravenously administered immunoglobulins were tried along with ulcer debridement with little response to treatment. Due to declining renal function and lack of response to apixaban, she was changed back to warfarin anticoagulation. During her last admission (6 months from the first presentation), she was found to have an infection by *Staphylococcus epidermidis* of her left shoulder joint prosthesis. The prosthesis was removed and she was commenced on vancomycin. This admission was further complicated by hemarthrosis of her left shoulder and rectal bleeding needing blood transfusions. A colonoscopy performed during this admission revealed bleeding hemorrhoids, which were managed conservatively. Due to recurrent bleeding and the need for repeated blood transfusions, a decision was made, after consultation with our patient, to stop anticoagulation.

## Discussion

Necrotic skin ulcers in association with a LA have been reported since 1963 [12]. However, non-vasculitic skin ulcers are an uncommon manifestation of APS and a recent study in France suggested that the prevalence of necrotic skin ulcers was only 3.5% in APS [9]. These skin lesions are usually painful, small, surrounded by a purple halo and purpura, and are located usually around the ankles. These ulcers are usually associated with livedo reticularis on the legs, which is the most common dermatologic manifestation of APS. Healing is usually associated with white atrophic scars with a dark pigmented halo resembling livedoid vasculitis [13]. The histopathology reveals fibrin deposition often with hyalinization within the walls and lumina of affected superficial vessels. Thrombi within the lumen and blood extravasation may be present but the absence of perivascular infiltrate excludes true vasculitis [3, 4]. In this patient, the skin biopsy findings excluded vasculitis as a cause of non-healing skin ulcers. Furthermore, laboratory evidence did not support a diagnosis of an active lupus as reflected by: normal complement levels, negative anti-dsDNA antibody, and absence of renal involvement as urine analysis was negative for active sediment. Our patient was compliant with anticoagulation as this was reflected by her therapeutic INR levels in the months prior to the development of skin ulcers. Livedoid vasculopathy associated with APS was considered a possible differential but was thought to be less likely due to the absence of immunoglobulin and complement deposits on immunofluorescence testing of biopsy samples. Finally, fulminant APS was considered a less likely diagnosis as this patient had no evidence of multiorgan failure with a rapid clinical deterioration.

### Differential diagnosis

The differential diagnosis of non-healing skin ulcers is broad. It includes warfarin-induced skin necrosis, although this usually occurs in the setting of protein C deficiency and usually develops within the first few days of starting warfarin [14]. Alternatively, calciphylaxis can present with necrotic ulcers although this is usually in the setting of end-stage kidney disease and is usually not seen with moderate chronic kidney disease, as in the present case [15]. Calciphylaxis can be diagnosed from calcium deposits on biopsy [16]. Vasculitis and pyoderma gangrenosum usually occur in the setting of inflammatory bowel disease although they can be seen in APS and can produce non-healing ulcers [17]. Other causes of non-healing ulcers in the context of APS can be due to non-compliance of anticoagulation, chronic disseminated intravascular coagulation (DIC), livedoid vasculopathy, and catastrophic APS [7]. Livedoid vasculopathy usually causes recurrent ulcers around both ankles which may heal slowly over 3–4 months with stellate white scars called *atrophie blanche* [18]. Livedoid vasculopathy may be associated with inherited thrombophilia, hyperhomocysteinemia, and APS [19]. Histopathology shows intraluminal thrombosis in 97.8% cases while direct immunofluorescence usually reveals immunoglobulins and complement components in blood vessels and the dermis [19].

Catastrophic antiphospholipid syndrome (CAPS) is a life-threatening variant of APS that is characterized by rapid development of arterial and/or venous thrombosis over a short period of time, usually ending in multiorgan failure [20]. Most episodes are precipitated by infections, surgery, or malignant diseases. In a majority of cases, small vessels are affected leading to a disseminated microangiopathic syndrome resembling thrombotic thrombocytopenic purpura [21]. The most frequently involved sites are kidney (73%), lungs (60%), brain (56%), and heart (50%) [22].

Prolidase deficiency is a rare autosomal recessive disorder, which presents with dysmorphic facies, mental retardation, splenomegaly, respiratory infections, and non-healing lower extremity ulcers [23]. Recent studies suggest that 10% of patients will develop SLE [24]. Prolidase deficiency was considered less likely in our patient as this condition usually becomes symptomatic between birth and 22 years of age [24].

Very few formal, prospective studies have evaluated the pathogenesis and treatment options for non-criteria manifestations of APS including any dermatologic lesions [25]. These manifestations often develop during, and may not improve with, heparin or warfarin anticoagulation [3]. Current treatment recommendations from the 13th International Congress on Antiphospholipid Antibodies Task Force, recommend that an INR of between 2 and 3 should be the target for patients who develop venous thrombosis associated with APS [26]. A higher target of 3.5 is recommended for patients who develop recurrence of thrombosis while on warfarin with an INR of between 2.0 and 3.0 [27].

It remains a mystery why this patient suffered from APS-related thrombosis after being stable a long time on anticoagulation. Studies have shown minimal likelihood of recurrence of thrombotic events in patients on moderate-intensity treatment with warfarin (with an INR between 2 and 3) [28]. We did exclude any new thrombotic risk factors as a cause for her microvascular thrombosis. In particular, occult malignancy was excluded in this patient.

Keeping in line with the evidence-based guidelines, we increased her anticoagulation intensity to maintain an INR between 3 and 4 but there was no significant improvement in her symptoms. Other treatment options were to switch from warfarin to therapeutic doses of unfractionated heparin or low molecular weight heparin but our patient refused to accept any injectable treatment options. Furthermore, she had an episode of bleeding per rectum with a significant drop in hemoglobin. We could not find any sinister cause for her bleeding apart from hemorrhoids which were managed with conservative measures.

Due to limited therapeutic options and reluctance on the part of our patient to switch over to injectable heparin, an informal decision was made to try apixaban. ODIs have a potential to be used in APS-related thrombotic events, although there is no concrete evidence supporting such use at the time of writing this report [29]. The available data from a phase 3 clinical trial suggest that using therapeutic fixed dose dabigatran or rivaroxaban may have a role in the management of patients with thrombotic APS [29]. Since approximately 10% of the patients who present with acute venous thromboembolism (VTE) have underlying APS, one can assume that ODIs will be effective in this category of patients as well [30, 31]. A recent randomized controlled trial has suggested that rivaroxaban is non-inferior to warfarin in patients with thrombotic APS (with or without SLE) [32]. However, this trial was limited to 6 months and it is unknown whether ODIs are effective in patients who develop recurrent thromboembolism while being on warfarin.

Apart from anticoagulation, isolated case reports exist describing other treatment options including the use of plasmapheresis and antifibrinolytics in some patients with APS [33, 34]. Other case reports have found successful treatment with the use of intravenously administered immunoglobulins [35], recombinant tissue plasminogen activator [34], rituximab [2], and sildenafil [36]. However, corticosteroids and cytotoxic agents have been found to be ineffective in APS [3].

## Conclusions

This case presents a rare late complication of APS-related microvascular thrombosis leading on to the development of non-healing skin ulcers despite adequate anticoagulation with warfarin. Currently, therapeutic options to treat recurrent thromboembolic events are limited in patients with APS. The efficacy of newer anticoagulants is still under investigation. In future, a better understanding of the mechanisms of aPL-mediated thrombosis may pave the way for the development of new therapeutic agents.

## Abbreviations

aCL: Anticardiolipin antibody
ANA: Antinuclear antibody
ANCA: Anti-neutrophil cytoplasmic antibody
aPL: Antiphospholipid antibody
APS: Antiphospholipid syndrome
Aβ2GPI: Anti-β2-glycoprotein I antibodies
CAPS: Catastrophic antiphospholipid syndrome
C-RP: C-reactive protein
CT: Computed tomography
D & C: Dilatation and curettage
DIC: Disseminated intravascular coagulation
dsDNA: Double-stranded deoxyribonucleic acid
ENA: Extractable nuclear antigen
GFR: Glomerular filtration rate
IIF: Indirect immunofluorescence
INR: International normalized ratio
LA: Lupus anticoagulant
ODIs: Oral direct inhibitors
SLE: Systemic lupus erythematosus
VTE: Venous thromboembolism
